# Whole-genome sequencing of multiple related individuals with type 2 diabetes reveals an atypical likely pathogenic mutation in the *PAX6* gene

**DOI:** 10.1038/s41431-022-01182-y

**Published:** 2022-10-07

**Authors:** Bernhard O. Boehm, Wolfgang Kratzer, Vikas Bansal

**Affiliations:** 1 Lee Kong Chian School of Medicine, Nanyang Technological University Singapore, Singapore, Singapore; 2 Department of Internal Medicine I, Ulm University Medical Centre, Ulm, Germany; 3 Department of Pediatrics, University of California San Diego, La Jolla, CA USA

**Keywords:** Disease genetics, Sequencing

## Abstract

Pathogenic variants in more than 14 genes have been implicated in monogenic diabetes; however, a significant fraction of individuals with young-onset diabetes and a strong family history of diabetes have unknown genetic etiology. To identify novel pathogenic alleles for monogenic diabetes, we performed whole-genome sequencing (WGS) on four related individuals with type 2 diabetes – including one individual diagnosed at the age of 31 years – that were negative for mutations in known monogenic diabetes genes. The individuals were ascertained from a large case-control study and had a multi-generation family history of diabetes. Identity-by-descent (IBD) analysis revealed that the four individuals represent two sib-pairs that are third-degree relatives. A novel missense mutation (p.P81S) in the *PAX6* gene was one of eight rare coding variants across the genome shared IBD by all individuals and was inherited from affected mothers in both sib-pairs. The mutation affects a highly conserved amino acid located in the paired-domain of *PAX6* - a hotspot for missense mutations that cause aniridia and other eye abnormalities. However, no eye-related phenotype was observed in any individual. The well-established functional role of PAX6 in glucose-induced insulin secretion and the co-segregation of diabetes in families with aniridia provide compelling support for the pathogenicity of this mutation for diabetes. The mutation could be classified as “likely pathogenic” with a posterior probability of 0.975 according to the ACMG/AMP guidelines. This is the first *PAX6* missense mutation that is likely pathogenic for autosomal-dominant adult-onset diabetes without eye abnormalities.

## Introduction

Diabetes mellitus is a very heterogeneous disorder that encompasses several distinct forms, each with characteristic clinical manifestations and age of onset [1–3]. Type 2 diabetes mellitus is the most common form of diabetes that results from a combination of genetic and environmental risk factors [1, 2] and is typically diagnosed after the age of 40 years. Maturity-onset diabetes of the young (MODY) is an autosomal dominant form of diabetes that typically occurs before 25 years of age in non-obese individuals and overlaps clinically with T2D [4]. Genetic studies of families using linkage mapping and candidate gene sequencing have identified more than ten genes that harbor highly penetrant disease mutations for MODY [5].

In recent years, genome-wide high-throughput DNA sequencing, particularly exome sequencing, has been extensively used to search for novel genes and variants that can cause monogenic diabetes [6–8]. For monogenic diabetes, exome-sequencing of families with multiple affected individuals has been used to identify pathogenic mutations using segregation analysis. A heterozygous missense mutation in the *WFS1* gene [7], loss-of-function mutations in the *APPL1* gene [9], and a missense mutation in the *MAFA* gene [8] have been shown to cause adult-onset familial diabetes using this strategy. Patel et al. [10] utilized a case-control strategy to show that protein-truncating variants in the *RFX6* gene cause monogenic diabetes, albeit with reduced penetrance compared to classical MODY.

Sequencing studies of large case-control cohorts for T2D have demonstrated that a small fraction of individuals diagnosed with T2D actually harbor pathogenic mutations in MODY genes [11, 12], highlighting the clinical and genetic overlap between T2D and MODY. This indicates that in the absence of a strong family history or due to late diagnosis of disease, some individuals with monogenic diabetes are clumped together with individuals with common T2D. It also suggests that genome-wide sequencing of individuals with T2D – particularly young-onset T2D – in combination with functional or family-based studies can identify novel variants with high-penetrance for adult-onset diabetes [13]. The starting point of this study was the discovery of a group of potentially related individuals – all diagnosed with T2D in a previous case-control sequencing study for T2D [11]. Since none of the four individuals carried a deleterious variant in a known MODY gene and two of the individuals were diagnosed with diabetes before the age of 40 years (31 and 37 years), we hypothesized that the diabetic phenotype of these individuals is mediated by novel high-penetrance risk variant(s). Therefore, we performed whole-genome sequencing (WGS) and searched for rare variants shared by the four individuals.

## Materials and methods

### Subjects

The four individuals selected for whole-genome sequencing were identified from a previous case-control sequencing study for T2D [11]. All individuals were residents of Germany and clinical data was provided by the treating physicians. All individuals were screened for the presence of diabetic neuropathy (examination of lower limbs). In addition, a yearly eye examination was performed on each individual by an experienced ophthalmologist as part of the diabetes disease management program in Germany (DMP diabetes). The examination included the quantification of intraocular pressure (using a non-contact tonometer), slit lamp examination, and fundus examination. Macular oedema and optic nerve morphology were defined using optical coherence tomography (OCT) examination (Heidelberg Engineering Spectralis or Zeiss Cirrus machines) every two years.

### Whole genome sequencing and variant calling

Whole genome sequencing was performed on genomic DNA of the four individuals by Novogene. DNA libraries were prepared using the NEBnext DNA Library preparation kit and sequenced using the Illumina sequencing technology and 150 base pair (bp) paired-end reads. Sequencing, alignment and variant calling metrics for the four individuals are reported in Supplementary Table 1. Sequence reads were aligned to the UCSC hg19 reference genome using the BWA aligner and PCR duplicates were removed using Picard (http://broadinstitute.github.io/picard/). Variant calling was performed jointly for the four individuals using the Genome Analysis Toolkit (GATK v4.0.1.2, HaplotypeCaller).

### Variant annotation and filtering

All identified variants were annotated using the Annovar annotation program and the RefSeq transcript database [14]. Missense variants were further annotated using in-silico prediction tools such as PolyPhen2 [15], SIFT [16] and Provean [17]. We also considered additional tools (FATHMM and MutationTaster) but these tools predicted all missense mutations in *PAX6* as deleterious and hence did not provide useful information. Variant allele frequencies were annotated using the human Genome Aggregation Database or gnomAD (v2.1,1) [18]. We prioritized variants predicted to have a functional impact on protein-coding genes (missense, splicing or loss-of-function) and with a maximum population allele frequency of less than 1%.

### IBD analysis

Genetic relatedness was estimated using the TRUFFLE tool [19] which also identifies identity-by-descent (IBD) segments for each pair of individuals from unphased genotype data. Pairwise IBD segments were intersected using bedtools [20] to identify regions that were shared IBD across all four individuals. For analysis of IBD on the X chromosome, we filtered out variants that were heterozygous in any of the three male individuals.

### PAX6 mutation analysis

The effect of the *PAX6* missense variants on protein stability was investigated in silico using the molecular modeling program FoldX [21]. FoldX was previously shown to be useful for identifying *PAX6* variants that disrupt folding or interactions with DNA [22]. For this purpose, the PAX6 protein structures for the DNA binding domain (6PAX) and homoedomain (2CUE) were downloaded from PDB and the fold command ‘buildModel’ was used to compare the free energy of the wild-type and the mutant residues. The difference in free energy (ΔΔG) averaged over five runs was used to assess the impact of each mutation on the protein stability. Information about PAX6 domain locations and protein sequences for PAX protein family was obtained from Uniprot (https://www.uniprot.org/). An image for the crystal structure of the paired-domain was obtained from the PolyPhen2 server [15].

### Data for association analysis

Variant data for *PAX6* (and *PER3*) in T2D cases and controls was obtained from the T2D Knowledge Portal (http://type2diabetesgenetics.org). Association tests for the *PER3* protein-truncating mutation were also conducted using the portal.

## Results

Using data from a recent case-control sequencing study of genes associated with monogenic diabetes in a sample of 6,888 individuals from Germany [11], we identified four individuals – all diagnosed with T2D – that shared multiple very rare variants (absent in large genomic databases). Such sharing is indicative of recent common ancestry and suggested that the individuals are related to each other. All four individuals also had a positive family history of diabetes - one parent and at least one sibling with T2D (Table 1). In addition, one of the four individuals was diagnosed with diabetes at the age of 31 and was estimated to have a 1 in 3 chance of being positive for MODY using a probability calculator [23]. None of the individuals harbored any rare deleterious variants in commonly mutated MODY genes (GCK, HNF1A, HNF4A, and INS). All individuals were non-obese (BMI ranging from 26.7 to 28.4 kg/m^2^), were negative for islet cell antibodies and GAD antibodies, had residual insulin and C-peptide levels, and were initially treated with oral anti-diabetic drugs (Table 1). All four individuals were also diagnosed with distal symmetric polyneuropathy, a diabetic complication that typically manifests after many years of diabetes mellitus [24]. The clinical presentation of neuropathy was uniform in all individuals with numbness in the feet, and burning pains in the legs present within three to five years following diabetes diagnosis. Examination of the lower limbs revealed sensory loss of vibration as well as temperature perception. Vitamin B12 deficiency, which can commonly be induced by the first-line oral diabetes treatment metformin, was ruled out in all subjects.

**Table 1:** Clinical phenotypes for the four individuals that underwent whole-genome sequencing.

ID	Gen-der	BMI	Age	AD	FG (mg/dl)	c-peptide (ng/ml)	Fasting insulin (uU/ml)	Family history of diabetes
I	F	26.7	54	37	146	2.5	35	Mother & one sibling
II	M	27.4	58	31	129	2.0	22	Mother & one sibling
III	M	28.4	71	42	147	1.6	13	Mother & one sibling
IV	M	27.4	62	59	142	2.8	33	Mother & two siblings

*DM* Diabetes mellitus, *AD* Age at which diabetes was diagnosed, *FG* Fasting glucose, *BMI* Body mass index in kg/m^2^_._

These phenotypes were recorded at the time when these individuals were recruited for the study of type 2 diabetes.

The strong family history of diabetes, early age at diagnosis for two of the four individuals, and the strong likelihood of relatedness between these individuals strongly indicated a monogenic diabetes phenotype driven by rare variant(s) with high penetrance. Hence we performed whole-genome sequencing (WGS) on the four individuals using paired-end (2 × 150 reads) Illumina sequencing technology (Methods). Sequencing generated 110–120 gigabases (Gb) of aligned sequence data (30–35x coverage) for the four individuals (Supplementary Table 1). Approximately 7.2 million variants (single nucleotide variants and short insertion/deletions) were identified in the four individuals relative to the reference human genome (hg19) using the GATK variant caller (Methods).

Identical-by-descent (IBD) analysis of the variants identified from the WGS data using the Truffle tool [19] confirmed that the four individuals are indeed related; individuals I and II shared 52.7% of the genome IBD, indicative of first-degree relatives. Since both these individuals reported an affected mother and an affected sibling, it is highly likely that they represent a sib pair. Similarly, individuals III and IV shared 41.3% of the genome IBD, and are also likely to be a sib pair. Furthermore, all other pairs (I-III, I-IV, II-III and II-IV) showed IBD sharing of 8–12% which is typical of third-degree relatives (Supplementary Table 2). Using the pairwise IBD segments, we identified genomic segments that were identical-by-descent (IBD) among all four individuals (Methods). Nine segments (average length of 21.6 megabases) that span 194.8 megabases (Mb) of DNA sequence were shared IBD among all four individuals (Supplementary Table 3).

**Fig. 1:**
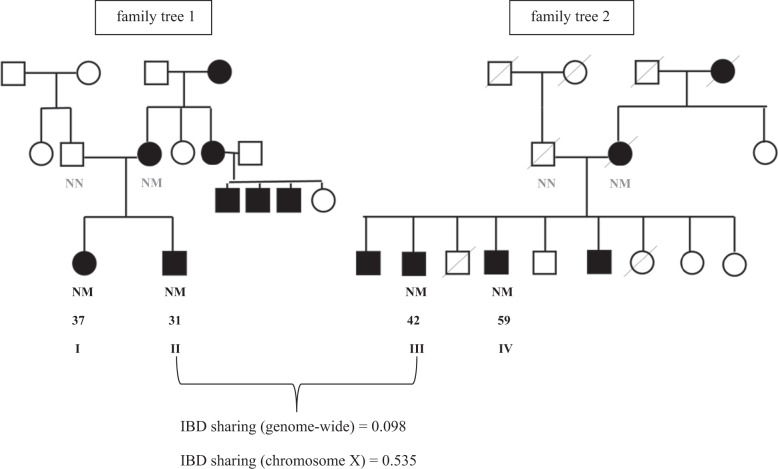
Family trees of the four sequenced individuals (I-IV) with diabetes that represent two sibling-pairs. Males are represented using squares and females by circles; a diagonal line indicates deceased individuals. Black filled symbols correspond to individuals with type 2 diabetes (DM). Data under the symbols represent the mutation carrier status (N = Normal allele, M = Mutation) and the age at diagnosis of diabetes (available only for the four sequenced individuals). The mutation status for the parents of individuals I-IV was inferred using IBD sharing on chromosome X.

Next, we contacted the treating physicians for these four individuals and were able to obtain detailed family trees which confirmed that individuals I & II (Family 1) and individuals III & IV (Family 2) represent sib-pairs (Fig. 1). The family trees revealed that individuals III and IV (Family 2) had two additional siblings diagnosed with T2D (4/9 siblings in total). In Family 1, there was a clustering of individuals with T2D on the maternal side. Notably, the diabetes phenotype was present in three generations and was consistent with dominant inheritance in both families. Although, the mothers of both sib-pairs were affected with diabetes, no data indicating that the mothers are related was available. Analysis of IBD sharing patterns on chromosome X revealed that individual II (from Family 1) and individual III (Family 2), both males, shared a 68.8 Mb segment of chromosome X IBD. None of the other pairs of individuals shared a segment of length 10 Mb or more on chromosome X. Since the single copy of chromosome X in males is maternally inherited, this indicated that the mothers of the sib-pairs in Family 1 and 2 are genetically related and the nine IBD segments shared by all four individuals are inherited from a recent common ancestor of the mothers of the two sib-pairs.

To search for genetic variant(s) that could explain the presence of diabetes in the two sib-pairs under a dominant model of inheritance, we analyzed the variants that intersected the nine IBD segments shared by all four individuals and filtered out common variants present at a minor allele frequency of 1% or greater in the gnomAD database (genome-wide variant data from > 140,000 individuals). Due to the large number of candidate variants (3439) that remained after this filtering, we prioritized variants located in protein-coding regions. There were only eight such coding variants (Table 2) including one stop-gain variant, five missense variants and two silent or synonymous variants. Only one of the eight coding variants (NM_000280:c.C241T:p.P81S) was absent in the gnomAD database and located in the *PAX6* gene that encodes for a transcription factor with important functions in the development of eye, nose, central nervous system and the pancreas. Since *PAX6* mutations have been observed to reduce insulin secretion in humans [25, 26] and the PAX6 protein is a key regulator of pancreatic islet development [27], the *PAX6* missense variant is a strong candidate for being a pathogenic variant. Among the other genes, only *PER3* had obvious functional relevance for diabetes since a tandem repeat polymorphism has been suggested to be associated with the risk of T2D [28] and Per3 knockout mice have altered body composition and glucose intolerance due to increased adipose mass [29].

**Table 2:** List of rare coding variants (allele frequency < 1%) shared IBD by all sequenced individuals.

Variant coordinates^a^	Gene	Variant annotation	Coding impact	gnomAD allele frequency^b^
11:31823225:G:A	PAX6	missense	NM_001310159: c.C241T:p.P81S	absent
1:7897090:G:T	PER3	stop-gain	NM_001289862:c.G3430T:p.E1144X	0.000012 (0.0000264)
16:22161180:G:A	VWA3A	synonymous	NM_173615:c.G3057A:p.A1019A	0.000021 (0.000083)
1:8557500:G:A	RERE	synonymous	NM_001042681:c.C969T:p.N323N	0.000057 (0.000085)
4:147179909:C:T	SLC10A7	missense	NM_001317816::c.G889A:p.A297T	0.004685 (0.009372)
5:132161522:G:A	SHROOM1	missense	NM_133456:c.C311T:p.P104L	0.00897 (0.01279)
6:139202137:T:C	ECT2L	missense	NM_001195034:c.T1709C:p.V570A	0.004216 (0.007084)
11:32119904:G:A	RCN1	missense	NM_002901:c.G457A:p.A153T	0.006932 (0.01048)

^a^The variant coordinates (chromosome:position:reference_allele:alternate_allele) are provided with respect to the hg19 human reference genome sequence.

^b^The overall allele frequency from gnomAD is reported along with the allele frequency in European (non-Finnish) population in brackets.

**Fig. 2:**
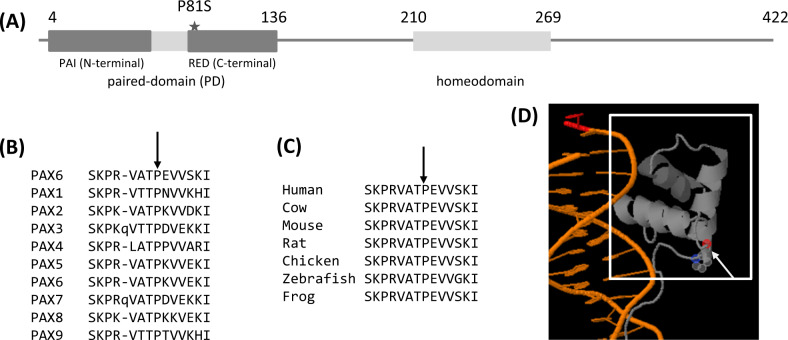
Location of the p.P81S missense variant in the sequence and the 3-D structure of the PAX6 protein. **A** Schematic of the PAX6 protein (422 residues long) with the two functional domains: paired domain (residues 4-136) and homeodomain (210-269). The p.P81S is located in the RED sub-domain of the paired domain. The proline at position 81 is conserved across all 9 members of the PAX protein family (**B**) and across the PAX6 protein sequences of several species (**C**). The crystal structure of the PAX6 paired-domain bound to DNA shows that the proline residue at position 81 (marked by an arrow) is the first residue of a helix in the RED sub-domain (**D**).

Both loss-of-function and missense mutations in *PAX6* are known to cause aniridia, a severe eye abnormality, as well as a range of congenital eye defects [30, 31]. The p.P81S mutation is located in the paired domain (PD) of the PAX6 protein that is involved in DNA binding (Fig. 2A, D) and is a hotspot for pathogenic missense mutations identified in individuals with eye abnormalities [32–34]. However, available clinical data did not indicate any eye-related abnormality in any of the four individuals. In addition, all individuals underwent an annual comprehensive eye exam as part of their diabetes management program (see Methods). The results (summarized in Supplementary Table 5) did not reveal any evidence of clinical features related to misdirected retinal development or those seen in individuals with congenital aniridia. Several studies have shown that *PAX6* protein-truncating mutations in aniridia pedigrees also co-segregate with diabetes and glucose intolerance [25, 26]. Additionally, isolated *PAX6* mutations have been reported to be associated with type 1 and type 2 diabetes (summarized in Supplementary Table 4). Therefore, we considered the possibility that this missense mutation is pathogenic for diabetes in the absence of eye abnormalities.

The *PAX6* p.P81S substitution is predicted to be deleterious for protein function by multiple in-silico tools such as PolyPhen2 [15] (probably damaging, HumDiv score = 0.97), SIFT [16] (deleterious, score = 0.00) and PROVEAN [17] (deleterious, score = −2.985). In addition, the P81 residue is completely conserved across multiple members of the PAX family of transcription factors in humans (Fig. 2B) and across PAX6 proteins in different species (Fig. 2C). We used the protein design tool FoldX [21] to analyze the impact of the P81S mutation on the PD domain of PAX6. FoldX has previously been shown to be powerful for modeling the impact of missense mutations on PAX6 function and can discriminate pathogenic PAX6 missense mutations from putative benign variants present in the gnomAD database [22]. Foldx modeling predicted that the variant had a ΔΔG (difference between folding free energy of mutant and wild-type) equal to 1.54 kcal/mol, slightly below the threshold of 1.6 kcal/mol used for classifying variants as “destabilize folding” [22]. The crystal structure of the paired-domain [35] showed that the p.P81S missense mutation does not affect a residue directly involved in DNA binding unlike most *PAX6* missense mutations that cause eye abnormalities.

Since *PAX6* is a well-established Mendelian disease gene, we used the ACMG/AMP guidelines for variant interpretation [36] to determine the pathogenicity of the missense variant. It satisfies two moderate criteria for pathogenicity (PM1 and PM2) and two supporting criteria (PP3 and PP4, see Table 3 for details). In addition, *PAX6* is highly constrained against missense variants, particularly in the paired-domain (6-fold reduction, see Supplementary Data). Therefore, the p.P81S mutation also satisfies the PP2 criteria (Table 3). Since the IBD segments shared by the two sib-pairs are maternally inherited and both mothers are affected by diabetes while the fathers are not, we can infer that the mutation segregates perfectly with disease status in the six affected and two non-affected individuals (see Fig. 1) and hence satisfies the PP1 supporting condition. Using the ACMG/AMP criteria to combine evidence types, this mutation can be classified as likely pathogenic. Using the Bayesian framework for combining relative odds of pathogenicity [37], we calculated the combined odds of pathogenicity for the p.P81S mutation to be 350.9 which translates to a posterior probability of 0.975 of being pathogenic.

**Table 3:** List of the ACMG/AMP criteria for pathogenicity satisfied by the p.P81S mutation in *PAX6* and the corresponding evidence supporting each criterion.

Category	Code	Description	Evidence
Moderate	PM1	Located in a critical and well-established functional domain without benign variation	located in the paired domain that plays a key role in DNA binding and is highly constrained for missense mutations
Moderate	PM2	Absent from controls in large population data	absent in gnomAD exomes (141,456 individuals)
Supporting	PP1	Co-segregation with disease in multiple affected family members in a gene definitively known to cause the disease	Both sib-pairs and their mothers (inferred) have diabetes and carry the mutation; PAX6 loss-of-function mutations have been shown to cause diabetes in aniridia families
Supporting	PP2	Missense variant in a gene that has a low rate of benign missense variation and in which missense variants are a common mechanism of disease	PAX6 has strong constraint against missense variants (gnomAD metric Z = 2.48), missense mutations are known to be pathogenic for a spectrum of eye abnormalities
Supporting	PP3	Multiple lines of computational evidence support a deleterious effect on the gene	Predicted to be deleterious by SIFT, Provean and Polyphen2, conserved across PAX transcription factor family, and predicted to destabilize protein using FoldX tool
Supporting	PP4	Patient’s phenotype or family history is highly specific for a disease with a single genetic etiology	Three-generation family with diabetes, one individual diagnosed at 31 years of age, family tree consistent with autosomal dominant inheritance of disease

We hypothesized that additional missense mutations in *PAX6* that cause monogenic-like diabetes in the absence of eye abnormalities exist in humans. Therefore we searched for such mutations in our previously published case-control sequencing study [11] and the T2D-GENES case-control dataset [38] that contains exome sequence data for more than 43,000 individuals. We restricted the search to missense mutations that shared three features with the p.P81S mutation: (i) predicted to be deleterious by multiple tools (SIFT, PolyPhen2 and Provean), (ii) conserved across the PAX family, (iii) and located in a functional domain. We identified one mutation in the Ulm-T2D dataset [11] and two missense mutations in the T2D-GENES dataset that satisfied these criteria (Table 4 and Supplementary Fig. 1). Each of the three mutations had a single carrier and each of the carriers was diagnosed with T2D. Two of the mutations from the T2D-GENES dataset had a single carrier in the gnomAD database. However, since the gnomAD database includes exome data from the T2D-GENES project, we can infer that both these mutations were absent in controls from gnomAD. Therefore, each of these mutations satisfies the ACMG/AMP criteria for being classified as likely pathogenic (PM1, PM2, PP2 and PP3). This provides additional evidence that a subset of missense mutations in *PAX6* likely cause T2D.

**Table 4:** Rare missense mutations in the *PAX6* gene identified in two case-control sequencing studies for type 2 diabetes that are (i) predicted to be deleterious by three missense prediction tools (PolyPhen2, SIFT and Provean), (ii) located in functional domains of the protein and (iii) constrained across the PAX protein family.

DNA change	AA change	dataset	gnomAD AF (control)*	Domain	FoldX ΔΔG (kcal/mol)	PolyPhen2 (HDIV)	SIFT score	Provean
c.37 G > C	p.G13R	T2D-GENES	0	paired	6.05	1.0	0.0	-6.69
c.284 C > T	p.P95L	T2D-GENES	0	paired	1.61	1.0	0.0	-8.21
c.708 T > A	p.D236E	Ulm	0	Homoe-box	1.43	1.0	0.014	-3.15

Reference sequences: *PAX6*, NM_000280.

*For estimating the gnomAD allele frequency, we subtracted allele counts for individuals from the T2D-GENES dataset.

Although we obtained compelling evidence that the *PAX6* missense variant is likely pathogenic, we explored the possibility that the *PER3* stop-gain variant (p.E1135X) could be pathogenic for the diabetic phenotype. This variant was observed in the gnomAD database with a very low allele frequency (0.000026) in European populations. Apart from a low-frequency non-synonymous variant (allele frequency of 0.59%) that was shown to causes an advanced phase sleep syndrome [39], no other coding variants in this gene have been associated with a human phenotype. Therefore, using ACMG/AMP criteria, the *PER3* stop-gain variant can be classified as a variant of unknown significance (VUS). Furthermore, we found a low-frequency frameshift variant (p.Q1169Kfs*29, rs771113980, allele frequency equal to 0.0013 in the Latino population) in this gene that is not associated with T2D (37 carriers, p-value = 0.079 in the T2D-GENES dataset) and is located near the p.E1135X variant. This further reduces the possibility that protein-truncating variants in *PER3* cause monogenic diabetes.

## Discussion

In this study, using whole-genome sequencing of four individuals that were ascertained from a large case-control study of T2D, we demonstrated that these individuals correspond to two sib-pairs and identified a novel missense mutation in the *PAX6* gene that is shared IBD by all four individuals. The mutation is predicted to be deleterious using conservation and other criteria, and co-segregates with the diabetes phenotype in six individuals. Using the ACMG/AMP guidelines for interpreting variants, we showed that this variant can be classified as likely pathogenic. Notably, available ophthalmological data ruled out the presence of aniridia or other ocular defects that are observed in individuals with PAX6 mutations.

The phenotypic spectrum of eye abnormalities of *PAX6* mutations is highly variable and ranges from classical aniridia (usually due to loss-of-function mutations) to mild ocular phenotypes such as nystagmus and microphthalmia [40]. Missense mutations are usually associated with milder phenotypes – in some cases even without iris defects [41]. Missense mutations have also been reported to cause phenotypes that affect only a subset of tissues with PAX6 expression. For example, studies of two Pax6 mouse missense mutants located in the paired domain (N50K and R128C) found that although both mutants have similar eye defects, the R128C mutant did not have gross craniofacial abnormalities [42]. Therefore, the detection of a likely pathogenic missense mutation in *PAX6* in a family with dominant diabetes without overt ocular phenotypes is not so surprising. Notably, a similar genotype-phenotype relationship has been observed for the *GATA6* gene. Mutations in this gene were first identified to cause congenital heart defects but subsequently missense mutations causing isolated adult-onset diabetes were also identified [43].

PAX6 is expressed in a number of cell types including lens, retina, brain and the pancreas and controls the expression of hundreds of genes in these cell types [44]. The expression pattern of PAX6 is consistent with the diverse eye defects [45], brain abnormalities [46] and diabetes observed in humans with *PAX6* mutations. Data from recent single-cell RNA-seq studies has shown that PAX6 is highly expressed in beta cells – both in the developmental stage and mature pancreas [47]. Furthermore, it is among the top five differentially expressed genes in the endocrine progenitor cell cluster along with other pancreatic developmental genes such as *PAX4* and *NGN3* [48]. Chromatin immumo-precipitation (ChIP) studies of PAX6 in lens and beta cells have shown that PAX6 binds the promoter and enhancer elements of thousands of genes and acts as a transcriptional activator and repressor [42, 44]. Therefore, both transcriptomic and chromatin-binding studies support the important role of PAX6 in the maintenance of beta cell function. A recent study [49] has shown that PAX6 regulates glucose-stimulated insulin secretion in human beta cells by modulating the expression of genes involved in exocytosis.

Although the co-occurrence of diabetes with aniridia was first reported in 2002 [25], few studies have systematically looked at the prevalence of diabetes in individuals with pathogenic PAX6 mutations. Wen et al. [26] performed a comprehensive evaluation of glucose metabolism in an aniridia pedigree with a stop-gain mutation and found a high penetrance of diabetes or IGT only in older individuals (8/8 individuals > 35 years of age versus 1/8 individuals < 35 years of age). More recently, a recent study [41] found that T2D was present in 12.8% of 86 patients with confirmed heterozygous *PAX6* mutations - twice the population prevalence. Although more studies are needed to determine the penetrance of diabetes in individuals with PAX6 mutations as a function of age and mutation type, based on the published studies (summarized in Supplementary Table 4), it is reasonable to infer that *PAX6* mutations do not typically cause young-onset diabetes but rather a slow progression from normal to impaired glucose tolerance which can manifest as diabetes later in life. Notably, the age at diagnosis of diabetes in the four individuals in our study is consistent with these observations.

All four individuals with the *PAX6* mutations had an early onset of chronic distal symmetric polyneuropathy. Peripheral neuropathy in diabetic subjects is quite common; a population-based survey in Germany found an overall prevalence of 42.2% with a significant increase in prevalence 25 years following T2D diagnosis [50]. Nevertheless, the early and unvarying appearance of distal neuropathy in all the carriers of the *PAX6* mutation suggests a potential common basis. However, the role of chronic hyperglycaemia as the main driver of the chronic distal symmetric polyneuropathy cannot be ruled out.

Our study represents a unique example of combining the power of large-scale case-control studies of T2D with family-based approaches. Previous studies have collected families with adult-onset multi-generation diabetes to search for novel genetic causes of diabetes [13]. In contrast, we were able to leverage related individuals identified from a case-control study. Since a subset of individuals were distantly related, only 6% of the genome was covered by IBD segments shared by all individuals and this significantly reduced the number of candidate rare variants. This strategy can be used to identify additional high-penetrance risk variants for diabetes and other common diseases that overlap with monogenic disorders. One limitation of our study is the presence of an ascertainment bias since additional individuals from the families were not available genotyping or phenotyping.

In conclusion, we have identified an atypical likely pathogenic missense mutation in the eye and islet transcription factor *PAX6* in a family with dominant adult-onset diabetes without overt eye defects. Analysis of large-scale case-control datasets shows that additional missense mutations in *PAX6* that are pathogenic for diabetes exist but are likely to be very rare since most deleterious missense mutations in *PAX6* cause developmental eye abnormalities, and hence are strongly selected against in human populations. Our results expand the spectrum of *PAX6* genotype-phenotype relationships from aniridia with diabetes to adult-onset diabetes without eye defects.

## Supplementary information

Supplementary Information

## Data Availability

The sequence data generated in this study is available from the corresponding authors on reasonable request.
