# Supplementary material for: Whole-genome sequencing of multiple related individuals with type 2 diabetes reveals an atypical likely pathogenic mutation in the PAX6 gene
Source: Eur J Hum Genet. 2022 Oct 7;31(1):89–96. doi: 10.1038/s41431-022-01182-y (PMC9823100; doi:10.1038/s41431-022-01182-y)

Supplementary Data

Estimation of missense constraint for PAX6

We fit a logistic regression model using variant data from the GnomAD database(25) (141,456 exomes) to estimate the deficit of missense mutations relative to synonymous or silent mutations in the *PAX6* gene(25). For each possible coding mutation (resulting in a missense or silent mutation) in the *PAX6* gene transcript (NM_000280), the predicted variable was the presence of the mutation in GnomAD database (0 or 1). The predictor variables included the tri-nucleotide mutation rate (to capture heterogeneity in mutation rates due to nucleotide context), the average coverage for the position in the GnomAD data, and the type of the mutation (0=missense, 1=silent). Mathematically,

$$P(Y_i = 1) = \text{logistic}\ (\alpha_0 + \alpha_1 \log (m_i) + \alpha_2\ c_i + \alpha_3\ t_i)$$

Here α's are the coefficients of the predictors in the logistic regression model, c_i is the average sequencing depth at the nucleotide position and t_i is the type of mutation. We can interpret α_3 as the log-odds of the mutation being present at a missense site compared to a silent site (controlling for other sources that influence the mutation).

The following table summarizes the constraint on missense variants in different regions of the *PAX6* gene:

**Region**	**AA range**	**Missense mutations**	**Beta**	**OR**	**p-value**
Full protein	1-422	114	-1.07	0.34	7.3E-18
Paired-domain	4-130	18	-1.92	0.15	1.6E-12
Paired-domain & homeodomain	4-130 & 210-269	20	-2.12	0.12	1.8E-16

Missense mutations in this gene show a strong deficit relative to the expected rate in the human population. In particular, missense mutations in the paired-domain, where the p.P81S mutation is located, show a more than 6-fold deficit compared to the expectation.

Supplementary Figures and Tables

Supp Table 1. Sequencing, alignment and variant calling statistics for the four individuals sequenced using Illumina WGS.

Individual	Sequenced bases (Gb)	mapped reads (%)	Average. depth	>= 10x coverage[a]	# of SNVs called (million)	dbSNP %[b]	Ti/Tv ratio[c]
I	109.00	99.78	36.72	98.66	3.723	98.68	2.04
II	104.40	99.80	35.18	99.11	3.708	98.66	2.04
III	103.91	99.79	35.05	99.13	3.706	98.75	2.04
IV	105.54	99.80	35.59	99.18	3.715	98.78	2.04

[a]percentage of genome covered by at least 10 aligned reads

[b]the number of SNVs that have been reported in dbSNP database (v141) divided by the total number of called SNVs

[c]the number of transitions divided by the number of transversions

Supp Table 2. Pairwise IBD sharing for the four individuals estimated using whole-genome sequence data and the Truffle IBD detection tool.

**ID1**	**ID2**	**p.IBD1 estimate**	**p.IBD2 estimate**	**IBD (fraction)**	**Relationship (degree)**
I	II	0.463	0.295	0.527	First
III	IV	0.457	0.185	0.413	First
I	III	0.205	0	0.103	Third
I	IV	0.239	0	0.120	Third
II	III	0.196	0	0.098	Third
II	IV	0.257	0	0.129	Third

Supp Table 3. List of genomic regions shared identical-by-descent by all four individuals. The nine regions cover a total of 194,810 kb of DNA sequence. The genomic coordinates are reported using the hg19 reference human genome sequence.

**Chromo-some**	**start (kb)**	**end (kb)**	**length (kb)**	**Number of genes**	**Diabetes-associated genes**
1	3,352	12,284	8,931	104	PER3
4	17,149	29,347	12,198	37	PPARGC1A
4	88,306	109,372	21,066	88	NFKB1, CISD2, SLC9B1
4	135,424	163,979	28,554	111	
5	10,532	31,601	21,069	32	GPR150, PCKS1
5	84,095	148,200	64,105	379	PAM
6	134,852	145,737	10,885	57	SLC35D3, PLAGL1
11	20,963	33,765	12,802	49	PAX6
16	12,613	27,811	15,198	131	

Supp Table 4. Summary of previous studies of individuals & families with aniridia (and PAX6 mutations) that examined diabetes-relevant phenotypes.

Reference	PMID	Mutation	Family	Metabolic phenotypes
Diabetes 2002	11756345	Stopgain (c.969C>T)	2-generation	Diabetes diagnosed at age of 22, co-segregation of aniridia with diabetes in two family members
		Frameshift/stopgain	Four individuals	4/4 individuals with aniridia had IGT (ages 28, 29,37, 54) but did not develop diabetes
Diabetologica 2009	19034419	Stopgain (c.1080C>T)	3-generation (19 member)	8/8 individuals > 35 years with aniridia also had diabetes or IGT, 6/7 individuals with aniridia and NGT were young (< 35 years)
Diabetes Res. & Clin.Prac. 2019	30572005	~484-630 kb distal deletion	2-generation	Co-segregation of aniridia and diabetes in all affected family members, 25 year male with IGT
Genetics in Epidemiology 2018	29850208	Frameshift (c.565TC>T)	4-generation	No diabetes in multiple individuals (5-31 years of age) except in one individual with T1D (diagnosed at 5 years of age)
JDI 2019	30151985	Frameshift ((c.483_486dupTTGG)	3-generation	Antibody-negative insulin-dependent diabetes diagnosed at 31 years of age, no diabetes in son at 26 years of age, T1D in daughter (35 years), mother with diabetes at older age
JDI 2015	25621140	Stopgain (c.969C>T)	Individual	Impaired glucose tolerance with impaired insulin secretion (at 40 years)
Diabetic Medicine 2005	15842522	Frameshift (c.402del2)	Individual	Non-autoimmune diabetes (age 15), low insulin secretory capacity, parents did not have diabetes or aniridia
Clinical & Exp. Opthamology 2013	23566044	Frameshift (c.1439delC)	5-generation (8 affected and 6 unaffected)	2/8 individuals with PAX6 mutation had IGT and one had diabetes, HbA1c in affected was higher than unaffected individuals (p=0.03), eye phenotype was different from aniridia and included ptosis, iris hypoplasia, and cataracts

GT = impaired glucose tolerance, NGT = normal glucose tolerance

Supp Table 5. Ophthalmological data for the four individuals with type 2 diabetes.

Individual	Visual acuity[a]	Intra-ocular pressure	Cataract	Diabetic retino-pathy[c]	Macular oedema[d]	Iris hypo-plasia	Nystagm-us	Optic nerve hypo-plasia[d]	Hetero-chromia[e]
I	R: 20/25<br>L: 20/25	Normal	No	No	No	No	No	No	No
II	R: 20/25<br>L: 20/25	Normal	No	NPDR[f]	No	No	No	No	No
III	R: 20/40<br>L: 20/50	Normal	Mild nuclear sclerosis	NPDR[f]	No	No	No	No	No
IV	R: 20/20<br>L: 20/25	Normal	No	No	No	No	No	No	No

[a]measured sing a Snellen chart: data from latest examination are reported – right eye (R), left eye (L)

[b]Intraocular pressure values were measured with noncontact tonometer (NCT); normal intraocular pressure values range between 10 to 21 mmHg

[c] classification of diabetic retinopathy was based on guidelines from Wilkinson CP et al., Ophthalmology 2003;110:1677–1682

[d]Macular oedema and optic nerve morphology were defined using optical coherence tomography examination (Heidelberg Engineering Spectralis OCT or OCT Zeiss Cirrus machines)

[e]three categories of heterochromia were excluded (complete heterochromia, central heterochromia and sectoral heterochromia)

[f]NDPR = non-proliferative diabetic retinopathy

Supp. Figure 1. Multiple sequence alignment of the proteins in the PAX transcription factor family in the paired domain (A) and homeodomain (B). For the paired-domain, 6 of the 9 proteins (PAX6, PAX1, PAX2, PAX5, PAX8 and PAX9) are shown while for the homoedomain, 4 of the 9 proteins in the family (PAX6, PAX3, PAX4, PAX7) that share this domain are shown. The multiple sequence alignment was obtained using the COBALT tool (https://www.ncbi.nlm.nih.gov/tools/cobalt/cobalt.cgi). Conserved amino acids are shown using red color. The three mutations (G13R, P95L and D236E) observed in case-control cohorts for type 2 diabetes that lie in the domains are highlighted with arrows.

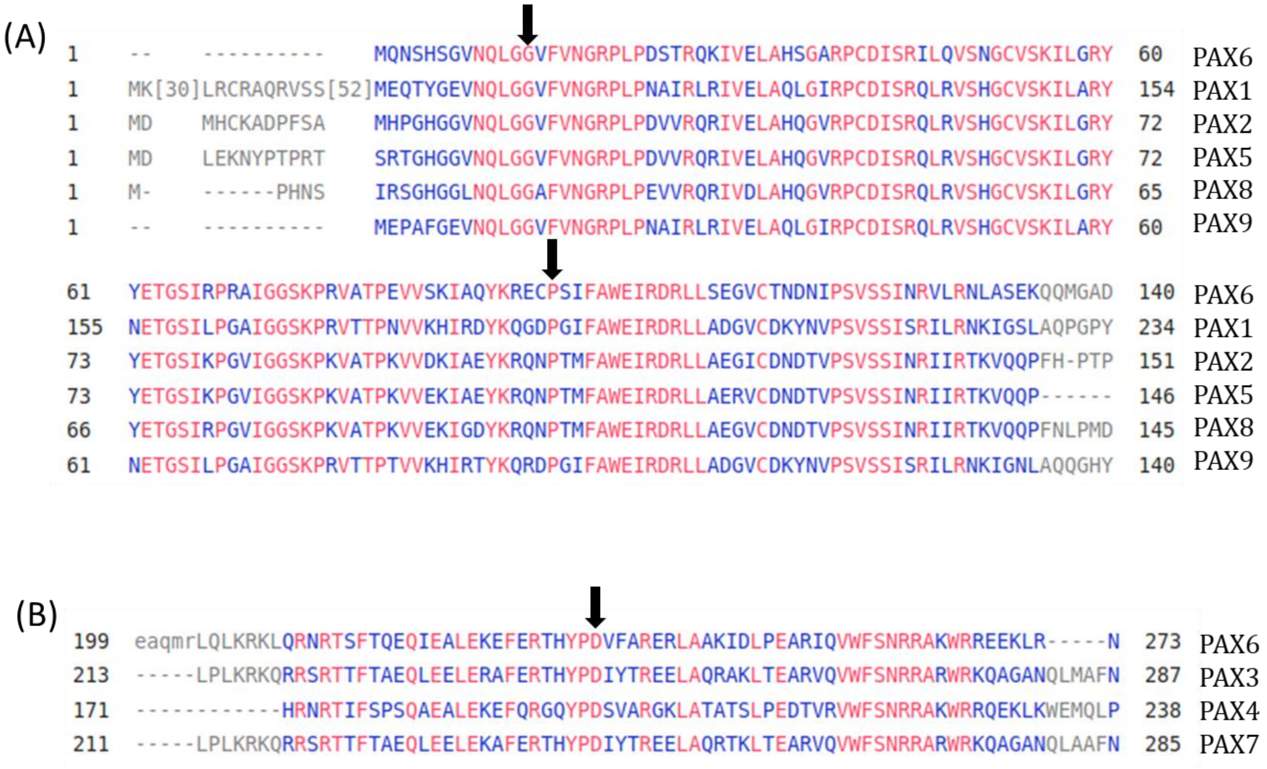

Supplement: Supplementary file 1 — Supplementary Information [file 41431_2022_1182_MOESM1_ESM.pdf]
